# A Magnetic Resonance-Compatible Fiberoptic Temperature Sensor for Measuring Focused Ultrasound-Induced Heating Without Artifacts

**DOI:** 10.1016/j.ultrasmedbio.2026.04.014

**Published:** 2026-05-15

**Authors:** Sara L. Johnson, Henrik Odéen, Harry Vine, Patricia Guenkawa, Benjamin Nelson, Allison Payne

**Affiliations:** a Radiology & Imaging Sciences, University of Utah, Salt Lake City, UT, USA; b OSENSA Innovations, Burnaby BC, Canada; c Biomedical Engineering, University of Utah, Salt Lake City, UT, USA; d School of Computing, University of Utah, Salt Lake City, UT, USA

**Keywords:** Focused ultrasound, Fiberoptic temperature probes, Temperature measurement validation, MRI temperature imaging, MR thermometry

## Abstract

**Objective:**

This work evaluated a fiberoptic probe that meets all features and qualities that render it highly suitable for validating magnetic resonance temperature imaging measurements for magnetic resonance-guided focused ultrasound (MRgFUS) applications. The fiberoptic probe performance was compared with other probes with viscous heating artifact (VHA) effects in both a free-field and a controlled tissue-mimicking phantom environment with MRgFUS heating applied.

**Methods:**

Three fiberoptic probes with different coatings (nylon, ethylene tetrafluoroethylene and polyimide) and tip diameters (1, 0.75 and 0.14 mm) were evaluated in free-field and tissue-mimicking phantom environments at 1 and 3 MHz under a variety of acoustic intensity and probe incidence angles with respect to the acoustic field. Concurrently acquired temperature measurements from the fiberoptic probes and magnetic resonance temperature imaging were compared, with Bland-Altman analysis also performed.

**Results:**

The 0.14 mm diameter fiberoptic probe composed of a glass optical fiber and ethylene tetrafluoroethylene coating had negligible VHAs under all acoustic exposure conditions. Comparison of the magnetic resonance temperature imaging and fiberoptic temperature data resulted in a root mean squared error of 0.52°C and 0.47°C and a limit of agreement of 1.2°C and 1.0°C when tested in a 1 and 3 MHz acoustic field, respectively. This contrasts with the results from a 1 mm-diameter fiberoptic probe composed of a polymer optical fiber with a nylon coating that demonstrated a root mean squared error and limit of agreement of 15°C and 3.5°C, and 37°C and 7.5°C at 1 and 3 MHz, respectively.

**Conclusion:**

The thin-diameter (0.14 mm) glass fiberoptic probe monitored focused ultrasound-induced heating in the acoustic field without evidence of a VHA. The ability to perform calibration and accuracy studies in an active acoustic field will broaden the range of testing allowed, improving the calibration range of magnetic resonance imaging temperature sequences.

## Introduction

Focused ultrasound (FUS)-localized hyperthermia and thermal ablation therapies to treat focal disease are often monitored in real time with temperature imaging. This allows both safety and efficacy to be assessed with quantitative temperature data. While ultrasound thermometry techniques have been investigated [1,2], magnetic resonance temperature imaging (MRTI) [3] is more commonly utilized in these procedures. Even when other bioeffects are utilized in FUS treatments including non-thermal ablation [4,5], histotripsy [6] or microbubble-driven blood–brain barrier opening [7,8], an accurate assessment of any treatment-induced temperature rise is often desired during therapy validation. While a non-invasive measurement technique is the goal of FUS temperature imaging, all temperature imaging methods must be calibrated and validated [9], often in different tissues and environments. This is typically achieved with invasive temperature probes that can be utilized in conjunction with the non-invasive measurement technique.

There are features and qualities that must be considered when selecting a temperature measurement probe for validating MRTI sequences. These include suitability for *in vivo* use, commercial availability, chemical stability, magnetic resonance imaging (MRI) artifact presence and viscous heating artifact (VHA) induced by FUS heating. Fine wire thermocouples (*i.e*., T and K-type) and fiberoptic probes [10] are often utilized as “ground-truth” point measures of temperature. Thin-film thermocouples have also been investigated [11,12], but while they do not exhibit the VHAs of other probes, they are not suitable for *in vivo* studies that require probe insertion. Thermocouples may also induce MRI artifacts that impact MRTI accuracy at the point of temperature measurement.

Fiberoptic temperature probes are very well-suited to the MRI environment and they have been used in several ways in MRI-guided FUS studies. They are used to validate MRTI measurements and can accurately assess the condition when the probe is placed outside of the ultrasound field [13–17]. Fiberoptic probes are also used in studies where the baseline temperature of the region of interest is required [18–21], or when a confirmation of temperature independent of the MRTI data is desired for safety or efficacy assessment [22]. Temperature probes used in conjunction with reference phantoms can also provide the information necessary to perform field drift correction, an essential element of longer duration magnetic resonance-guided focused ultrasound (MRgFUS) sonications [23].

All types of temperature probes can have artifactual heating caused by the interaction of the viscous forces from the FUS beam and the surrounding tissues. This VHA is a function of probe size, the construction material, orientation in the ultrasound field and other factors [24]. In terms of probe size, even small probes can have a non-negligible effect on the ultrasound field. Probes with the relationship $\frac{1}{2}\sqrt{D}>\lambda$, where D is the probe sensor diameter and *λ* is the FUS wavelength in the tissue, can scatter and reflect the ultrasound waves [24]. The VHA itself cannot be neglected, as studies have shown that up to 80% of the measured temperature rise is due to the VHA [12]. The VHA typically persists during FUS exposure and for milliseconds to seconds into the cooling period after the FUS exposure is turned off. This artifact has been quantified for different materials [12], and correction methods have been proposed that are based on detailed material characterization [25] or simulation techniques [26]. However, VHA effect-correction methods have not been validated with alternate ground-truth temperature measurements.

A fiberoptic temperature probe that could be used to validate temperature sequences for FUS technologies, without being affected by VHA, would be a highly valuable resource for the FUS community. While temperature imaging techniques have been successfully validated when the probe is in the FUS beam path [10], typically only data acquired when the FUS beam is off are utilized in the data analysis. This limits the amount of data included in validation and prevents accurate ground-truth measurements of peak heating in FUS exposures. This work evaluated a fiberoptic probe that meets all of the features and qualities that render it highly suitable for validating MRTI sequences for MRgFUS applications. The fiberoptic probe performance was compared with other probes with VHA effects in both free-field and controlled tissue-mimicking phantom environments with MRgFUS heating applied.

## Materials and methods

Three types of fiberoptic temperature probes were evaluated, as detailed in Table 1. All three probes (PRB-140, PRB-500 and PRB-G40) were from the same manufacturer, OSENSA Innovations (Burnaby, BC, Canada). This particular fiberoptic temperature measurement system was selected for its established compatibility in the MRI environment. The system is immune to radiofrequency coil emissions, gradient-induced electromagnetic interference and static magnetic fields, thereby minimizing measurement artifacts and temperature offsets associated with electromagnetic coupling. The system also employs phosphor-based fluorescence thermometry. Temperature is measured from the temperature-dependent fluorescent excitation and decay characteristics of the phosphor material at the probe’s distal tip. Following pulsed optical excitation from a light-emitting diode, the phosphor emits fluorescence that is detected by a photodetector. The system electronics analyze the temporal decay profile of the emitted signal, which varies predictably with temperature. Proprietary digital signal processing enables stable, low-noise signal acquisition, providing a temperature measurement accuracy of up to ±0.1°C under controlled conditions. Probe selection was based on fiber temperature rating, outside diameter, optical wavelength compatibility, material response to acoustic exposure within the FUS field, and mechanical durability. Prior laboratory testing indicated that minimizing the volume of material within the FUS field reduces probe self-heating and measurement bias.

All three chosen probes were tested under acoustic free-field testing, while only two probes were evaluated with MRgFUS in tissue-mimicking phantoms. Testing was performed over a range of acoustic intensities and probe incidence angles with respect to the acoustic field using two transducers (a 256-element phased array: f = 1 MHz, focal length = 13 cm, f-number = 0.84, free-field pressure FWHM = 1.7 × 1.7 × 7.3 mm^3^; and a 16-element phased array: f = 3 MHz, focal length = 3.5 cm, f-number = 0.7, free-field pressure FWHM = 0.52 × 0.52 × 2.25 mm^3^; both transducers are manufactured by Imasonic, Inc., Voray-sur-l’Ognon, France).

### Acoustic free-field testing

**Figure 1.**
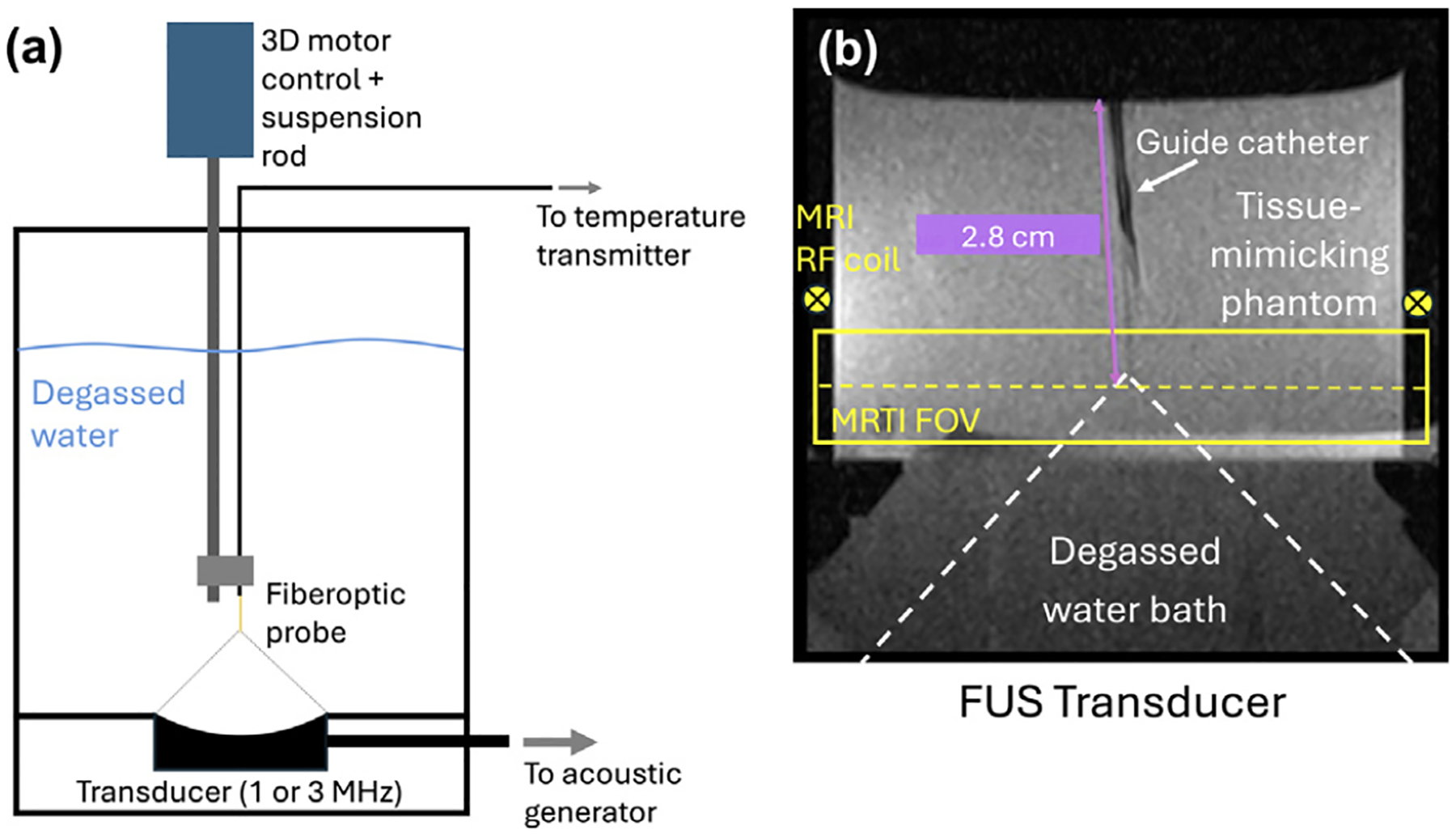
Experimental setups for acoustic free-field and tissue-mimicking phantom magnetic resonance-guided focused ultrasound (MRgFUS) testing. (a) Free-field testing was done in a vertical testing column filled with degassed water. The fiberoptic probe was positioned *via* a support rod and custom holder that was connected to a 3-D motor system. (b) An axial T1-weighted magnetic resonance image (MRI) showing the MRgFUS setup indicating the focused ultrasound (FUS) transducer position in relation to the tissue-mimicking phantom, fiberoptic probe and magnetic resonance temperature imaging (MRTI) field of view (FOV). A single-loop MRI radiofrequency coil encircles the phantom at the approximate level of the fiberoptic probe location.

The fiberoptic temperature probes (OSENSA Innovations) were evaluated under free-field conditions in degassed water to evaluate the heating artifacts caused by effects independent of the surrounding material or tissue. Each fiberoptic probe was placed in a custom mounting device (Fig. 1a), such that the fiberoptic sensor was positioned at the center of the focal spot of a FUS transducer at a 180° incidence angle using both the 1 and 3 MHz transducers. Fifteen second sonications were applied at increasing intensities (250–4100 W/cm^2^; Table 1), with temperatures recorded continuously at a 0.5 Hz frequency. The probes in Table 1 (PRB-500, PRB-140 and PRB-G40) were evaluated sequentially.

### Tissue-mimicking phantom MRgFUS testing

Probe measurement accuracy was evaluated in tissue-mimicking homogeneous phantoms during MRgFUS sonications monitored with magnetic resonance temperature imaging. The experimental setup is described in Figure 1b with an MRI T1-weighted axial image of the setup. This case was with a probe placed at a 180° incidence angle to the FUS beam. The phantoms were constructed with an 11% gelatin formulation in a 1:1 water-to-evaporated milk formulation [27]. Through-transmission measurements [28] were made of the phantom to characterize acoustic attenuation and speed of sound. Both the PRB-500 and PRB-140 probes were evaluated using the incident angles and acoustic intensity ranges detailed in Table 1, with each incident angle and acoustic intensity combination evaluated through n = 3 FUS sonications. MRTI measurements were obtained with a 3-D segmented-echo planar imaging gradient echo sequence (1 × 1 × 2 mm^3^ voxel size interpolated to 0.5 × 0.5 × 1 mm^3^ spatial resolution, 3.52 s per volume acquisition time, repetition time (TR) = 26 ms, echo time (TE) = 11 ms, flip angle = 12°, field of view = 128 × 128 × 20 mm, echo train length = 7) with the imaging volume centered at the position of the fiberoptic probe. The MRTI sequence used has been previously validated for accuracy using fiberoptic probes [10], where only FUS “off” data were used to evaluate precision and accuracy due to the VHA of the fiberoptic probe used for validation. Because the proton resonance frequency technique was used to calculate temperature difference, the fiberoptic probe temperature was used as the phantom baseline temperature for all MRTI measurements. All MRIs were done on a 3T MRI scanner (PrismaFIT, Siemens Healthineers, Erlangen, Germany) using an in-house–built, single-channel radiofrequency receive-only coil around the phantom.

**Figure 2.**
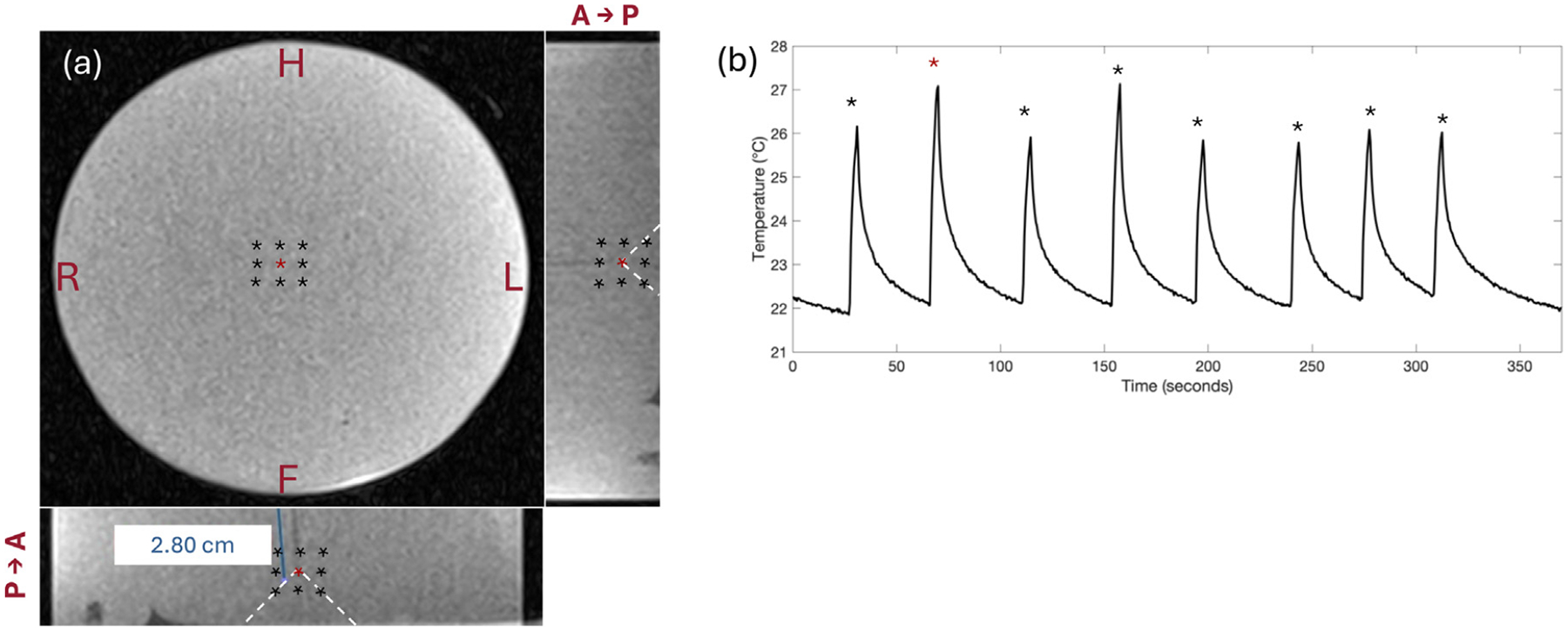
Schematic and fiberoptic temperature probe results detailing the fiberoptic probe location determination procedure in the tissue-mimicking phantom. (a) Determining the approximate probe location using a high-resolution T1-weighted image. All three orthogonal views are shown. Probe location refinement of was achieved through iterative 3 × 3 × 3 grid scanning at 0.25 mm step size with the focused ultrasound beam (illustrated grid is not to scale). The approximate acoustic field cone is overlaid with dashed lines. (b) The maximum temperature response while scanning through nine grid points. The maximum temperature response (red *) corresponds with true probe location.

In all cases, the fiberoptic probe location in the tissue-mimicking phantom was visualized with a high-resolution, T1-weighted MRI (Fig. 2a, volumetric interpolated breath-hold examination, 0.4 × 0.4 × 0.6 mm^3^ voxel size interpolated to 0.2 × 0.2 × 0.3 mm^3^ voxel spacing, TR/TE = 7.05/1.86 ms, flip angle = 7°, field of view = 128 × 128 × 26.4 mm^3^). Due to the size of the probe tip compared with the MRI voxel size, the probe tip location was verified by electronically steering the FUS beam in a 3 × 3 × 3 grid (0.25 mm spacing, 250 W/cm^2^ for 4 s/point with 30 s cooling between sonications) until the maximum fiberoptic temperature rise was measured (Fig. 2b). The location of maximum temperature rise was identified as the fiberoptic probe sensor location. After fiberoptic probe tip location determination, 18.6 s MRgFUS sonications at the acoustic intensities detailed in Table 1 were applied at the fiberoptic probe tip location, while both fiberoptic probe (0.5 Hz sampling rate) and MRTI measurements were acquired concurrently.

### Data analysis

The fiberoptic probe temperature data were temporally averaged to match the sample rate and recorded clock times of the MRTI acquisitions. Correlation between the paired MRTI and temporally averaged fiberoptic probe measurements was made both during the FUS-on and -off conditions. Bland-Altman analysis was also performed. The correlation coefficient, root mean squared error (RMSE) and limits of agreement (LOA) were calculated for all measurements.

## Results

### Acoustic free-field testing

**Figure 3.**
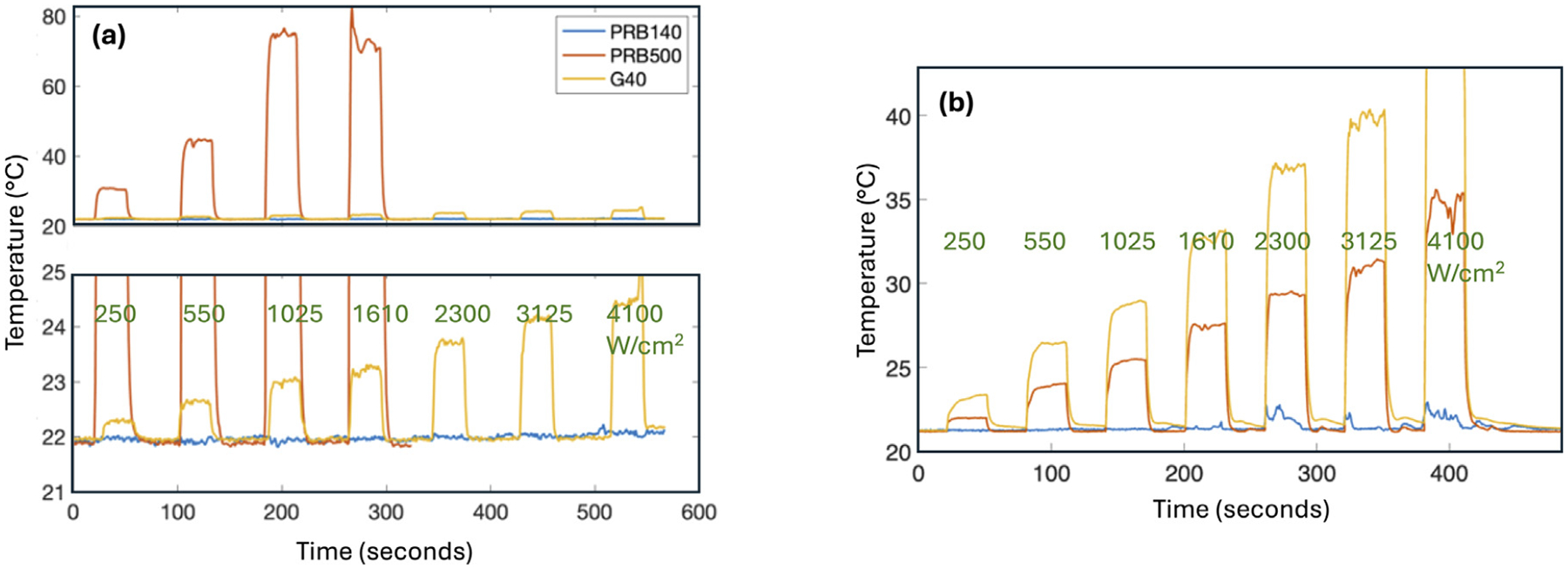
Acoustic free-field probe response at increasing acoustic intensities at both 1 and 3 MHz. (a) Fiberoptic probe response at 1 MHz transducer frequency. The top and bottom rows are equivalent but displayed with different y-axis scaling to better display the G40 probe response. Measurements for the PRB-500 probe were only obtained up to 1610 W/cm^2^ due to the magnitude of the viscous heating artifacts. (b) Response at 3 MHz frequency. The acoustic spatial peak intensity (I_sp_) applied during each 30 s sonication is indicated on each peak (*green*).

Figure 3 shows the performance of the evaluated fiberoptic probes in a non-absorbing medium (degassed water) over a range of applied acoustic intensities for both the 1 and 3 MHz transducers. At 1 MHz, the PRB-500 probe had the most pronounced VHA. Testing for that probe was halted after 1610 W/cm^2^ exposure intensity due to the severity of the artifact. The PRB-G40 probe had a modest VHA that increased linearly with acoustic intensity. The largest artifact manifested in an approximate 2.5°C temperature rise at the highest evaluated intensity of 4100 W/cm^2^. In contrast, the temperature measured by the PRB-140 probe was artifact-free and consistent across all evaluated acoustic intensities. At 3 MHz, as seen in Figure 3b, the PRB-G40 probe had the highest VHA, with increasing errors at higher spatial peak intensities. The PRB-500 probe also had large VHA errors of approximately half the magnitude of the PRB-G40 probe. There were very minor artifacts seen by the PRB-140 probe at spatial peak intensities greater than 2300 W/cm^2^, resulting in an apparent artifactual temperature rise of approximately 1°C.

### Tissue-mimicking phantom testing

**Figure 4.**
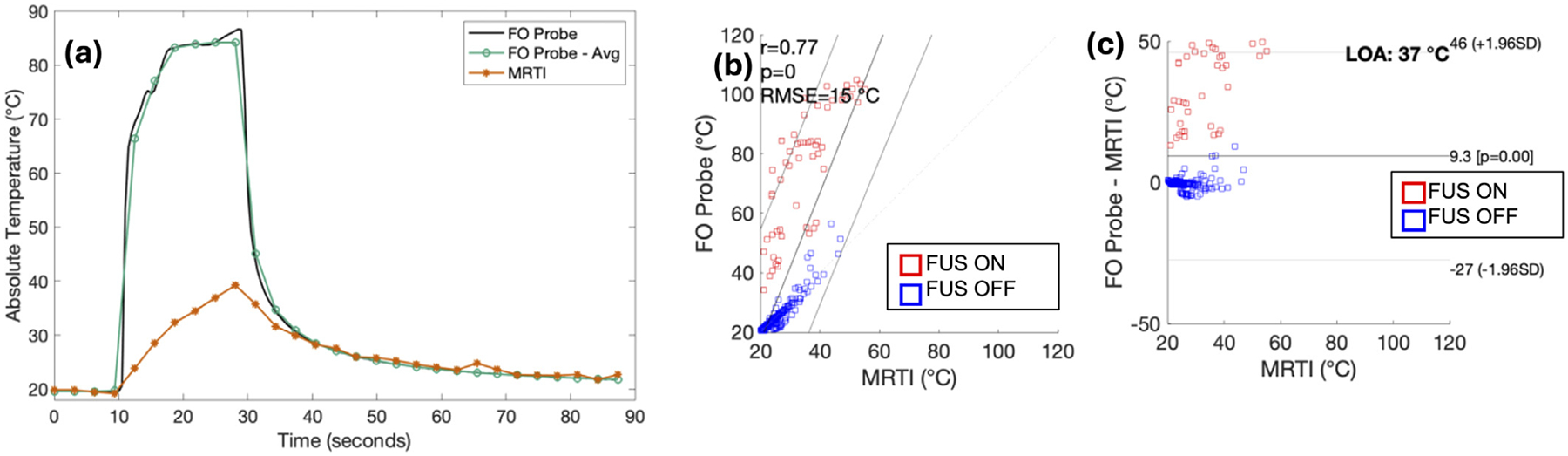
PRB-500 fiberoptic (FO) probe and magnetic resonance temperature imaging (MRTI) response for a 550 W/cm^2^, 18.6 s magnetic resonance-guided focused ultrasound (MRgFUS) sonication at 1 MHz. (a) Comparison of temperature versus time for the FO probe and MRTI data. Both acquired (*black*) and temporally averaged data (*green circles*) are shown compared with MRTI (*orange circles*). (b) Linear correlation and (c) Bland-Altman plots showed good agreement when the FUS was off, but the FUS-on condition had large errors, with an RMSE of 15°C and an LOA of 37°C. The FO probe incidence angle to the MRTI was 180°.

**Figure 5.**
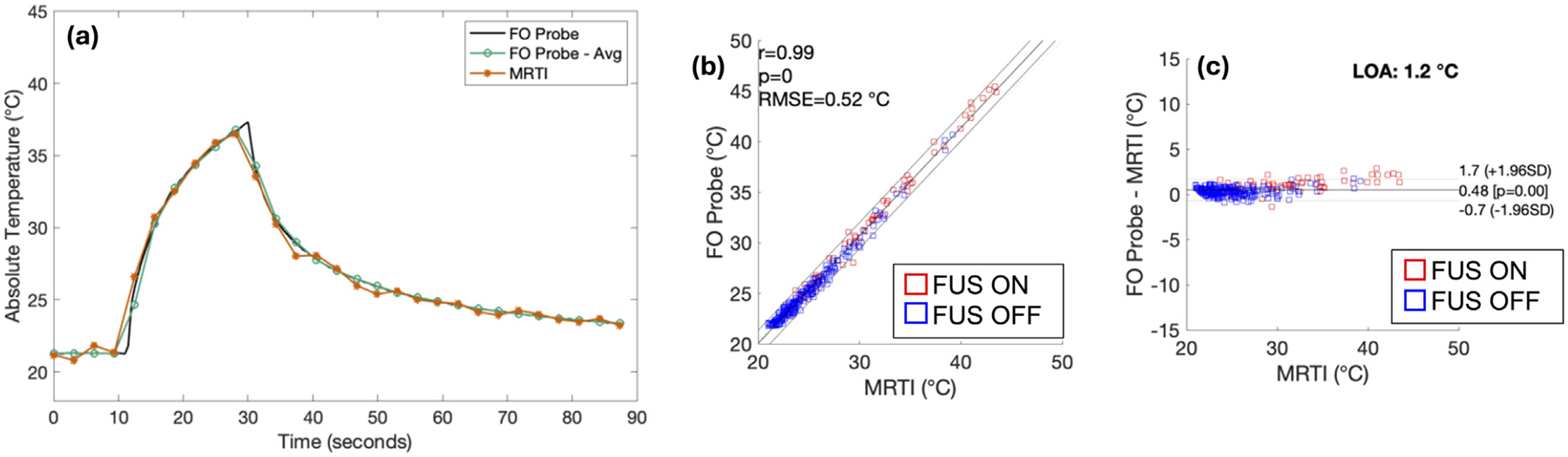
PRB-140 fiberoptic (FO) probe and magnetic resonance temperature imaging (MRTI) response for a 550 W/cm^2^, 18.6 s magnetic resonance-guided focused ultrasound (MRgFUS) sonication at 1 MHz. (a) Comparison of temperature versus time for the FO probe and MRTI data. Both acquired (*black*) and temporally averaged data (*green circles*) are shown compared with MRTI (*orange circles*). (b) Linear correlation and (c) Bland-Altman plots showed good agreement with both FUS-off and -on conditions, with an RMSE of 0.52°C and an LOA of 1.2°C. The FO probe incidence angle to the MRTI was 180°.

The acoustic properties of the phantom were measured to be 0.54 dB/cm and 1549 m/s for acoustic attenuation at 1 MHz and sound speed, respectively. Figure 4 shows the peak MRTI response in the gelatin phantom when exposed to a 550 W/cm^2^, 18.6 s sonication assessed with the PRB-500 fiberoptic probe. Both the original and temporally averaged fiberoptic probe temperatures are shown. The VHA was readily apparent in the PRB-500 probe measurement, with the probe measuring a temperature of approximately 85°C while the MRTI only measures a peak temperature of 40°C. Correlation and Bland-Altman analysis for the PRB-500 probe showed good agreement with MRTI when the FUS was off (blue marker), but there were errors up to 50°C when the FUS was on (red marker). Errors during the FUS-off condition were greater at higher temperatures, as the VHA artifact impacted the accuracy of the PRB-500 probe at the start of the cooling curve (~10 s). The RMSE across the entire sonication was 15°C, with a LOA of 37°C. Figure 5 shows the same analysis for the PRB-140 probe under equivalent sonication conditions. This probe exhibited minimal VHAs and a high correlation with MRTI (r = 0.99), an RMSE of 0.52°C and an LOA of 1.2°C. Similar studies were performed for the 3 MHz condition. The PRB-500 probe had an RMSE of 3.5°C with an LOA of 7.5°C, while the PRB-140 probe had an RMSE of 0.47°C with an LOA of 0.99°C.

**Figure 6.**
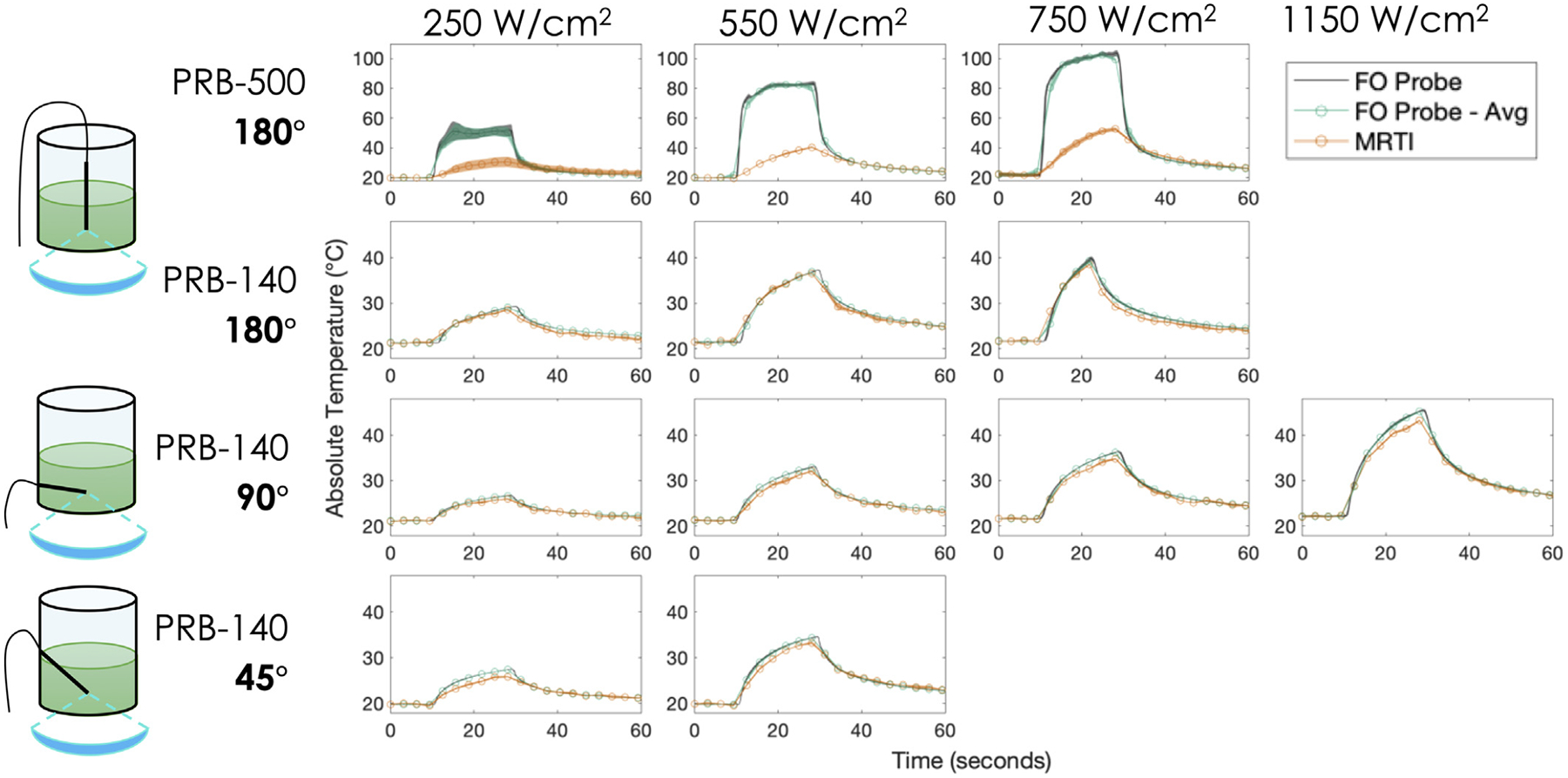
Comparison of PRB-500 and PRB-140 probe temperature measurements for magnetic resonance temperature imaging (MRTI) across a range of acoustic intensity (*columns*) and probe incidence angles (*rows*) performed at 1 MHz. The shaded area in all curves represents the one standard deviation of the N = 3 focused ultrasound sonications that were performed at each incidence angle and acoustic intensity combination. Both acquired (*black*) and temporally averaged data (*green circles*) are shown compared with MRTI (*orange circles*).

**Figure 7.**
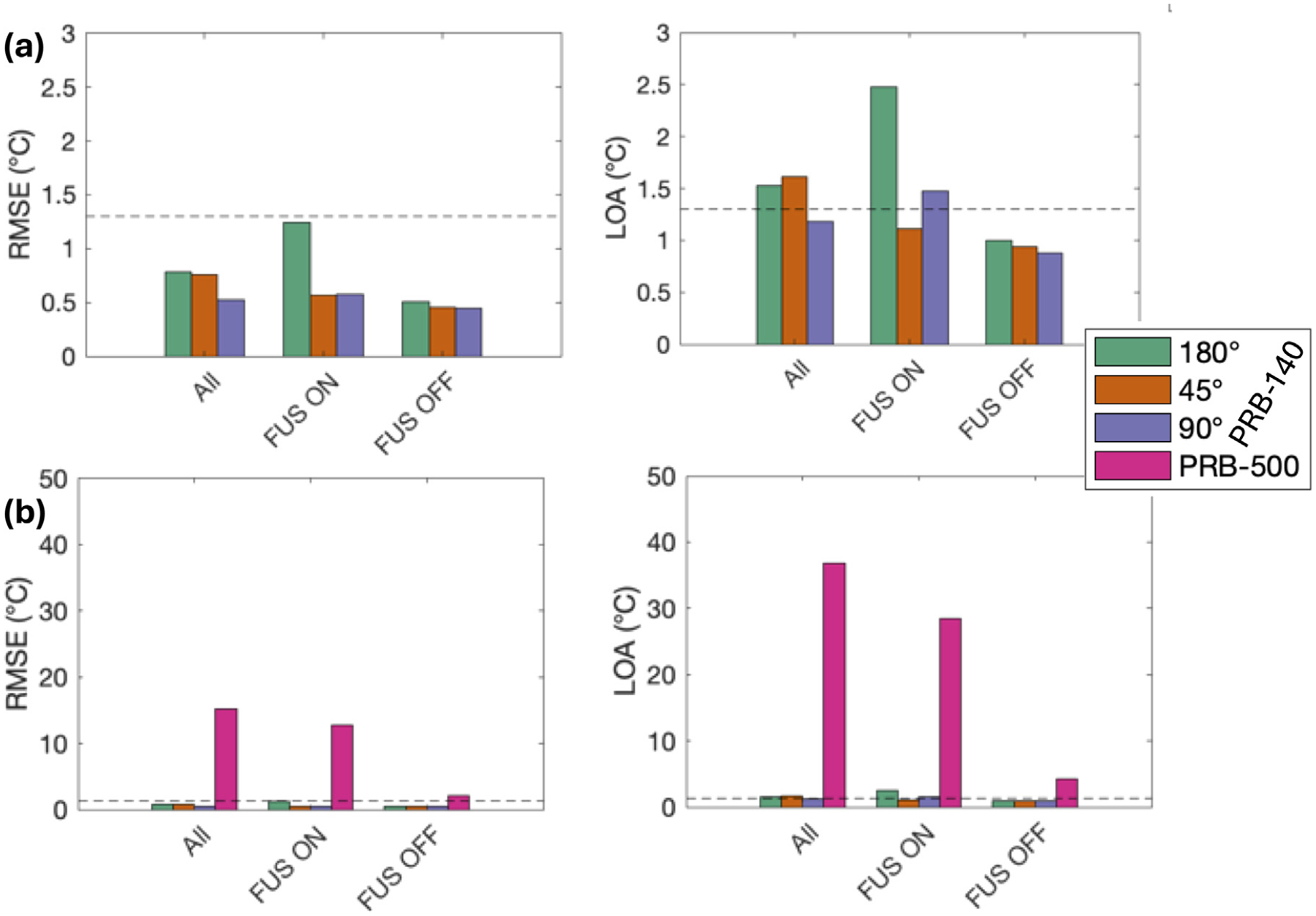
Root mean squared error (RMSE) and limits of agreement (LOAs) for the PRB-140 and PRB-500 fiberoptic probes for a 550 W/cm^2^ focused ultrasound (FUS) sonication performed at 1 MHz. Results for the PRB-140 probe are shown in (a), while (b) includes both PRB-140 and PRB-500 probe data. The dashed black line compares the RMSE and LOA with previously published accuracy data for the utilized magnetic resonance temperature imaging pulse sequence (1.3°C) [10]. Three incident angles were evaluated for the PRB-140 probe, while only the 180° condition was evaluated for the PRB-500 probe.

Figure 6 demonstrates similar results across both acoustic intensity and incidence angle between the probe and ultrasound beam when exposed to a 1 MHz acoustic field. While increasing acoustic intensity exacerbated the VHA in the PRB-500 probe similar to the free-field testing condition, both incident angle and acoustic intensity variables had minimal effects with the PRB-140 probe. Figure 7 quantifies the RMSE and LOA between the MRTI and fiberoptic probe measurements for the 550 W/cm^2^ sonication for the PRB-500 and PRB-140 probes, and across all incident angles for the PRB-140 probe. Figure 7a shows a narrower y-axis range and only includes PRB-140 probe results, while Figure 7b displays a wider y-axis range and includes both PRB-140 and PRB-500 probes. In general, RMSE and LOA were larger for a 180° incidence angle.

## Discussion

Fiberoptic temperature probes have been utilized extensively in FUS studies. While typically not used in clinical applications [29] due to the invasive nature of the probes, their use in temperature measurement validation, technique calibration and gold standard measurement is invaluable. The VHA experienced by most probes is influenced by a combination of probe tip materials, probe acoustic properties and the probe tip diameter. All probes utilized in this study had a probe tip diameter of ≤1 mm and therefore did not scatter or reflect the FUS beam at the frequency of the FUS beam. However, probe diameter could contribute to the overall VHA observed. Early VHA studies indicated that errors due to the thermal properties of the probe materials were negligible when probe thickness was on the order of 1/20th of a wavelength or less [12]. Probe material may have also contributed to the VHA, which can arise from a mismatch in density between the probe and the surrounding medium [12]. Only fiberoptic probes were evaluated in this study due to their compatibility with the MRI environment, and the evaluated fiberoptic probes were constructed with ethylene tetrafluoroethylene probe material. This material choice was specifically selected by the manufacturer for ultrasound applications due to its wide operating temperature, chemical inertness, and options for biocompatibility. Other considerations included small fiber diameter, flexibility, relatively low absorption of ultrasonic energy (therefore low heating factor), low thermal conductivity, and high commercial availability. Other temperature probe types including fine wire [30] and thin-film thermocouples [12] have been studied in other work [24,31].

Both the PRB-500 and PRB-140 probes have good correlation with the MRTI measurement when the FUS field is off, as has been demonstrated in other work that used a different fiberoptic probe (400 *μ*m sensitive area, Neoptix T1 with polyimide cap; Qualitrol, Fairport, NY, USA) [10]. However, the PRB-500 probe still had a higher RMSE and LOA when compared with the equivalent PRB-140 probe condition when the ultrasound field was off. The PRB-500 probe RMSE and LOA were 2.14°C and 4.60°C, respectively, at the 180° incidence angle, compared with 0.51°C and 1.0°C for the PRB-140 probe at 1 MHz. Similar trends were seen with a 3 MHz transducer frequency, with the PRB-500 probe having a RMSE and LOA of 0.74°C and 1.60°C when the ultrasound field was off. Only the PRB-140 probe had good correlation during the FUS-on condition, with a mean RMSE and LOA over all incidence angles of 0.79°C and 1.69°C, respectively, at 1 MHz, and with a 0.48°C and 1.2°C RMSE and LOA, respectively, at 3 MHz. These results comparing MRTI and probe agreement while FUS is on are within the range of previously reported accuracy of the MRTI sequence of 1.3°C, which was measured using a fiberoptic probe with apparent VHA artifact using the “wait-and-see” method after the FUS was turned off [10]. The accuracy reported in this study with the RMSE at 0.79°C was also within the range of prior reporting.

One of the key difficulties in MRTI validation studies with fiberoptic probes is difficulty locating the probe tip on MRI. In this study, a location protocol was implemented that utilized both visual analysis of high-resolution 3-D T1-weighted images as well as interrogating the probe position with fine-resolution FUS beam targeting. Visual analysis was performed on an image with an interpolated resolution of 0.2 × 0.2 × 0.3 mm^3^. While this resolution is adequate for the 1 mm probe diameter (PRB-500), it is more difficult to locate the PRB-140 probe, which has a probe tip of 0.14 mm. We found that a grid search (3 × 3 × 3 pattern with 0.25 mm spacing) with the FUS beam allowed the probe position to be refined (Fig. 2b). An improvement in MRI visibility would allow for more accurate spatial targeting. However, for *in vivo* studies, these high resolutions can be challenging to achieve in a reasonable scan time, especially on clinical MRI scanners, and the grid search approach can be a relatively quick and easy approach to accurately locate the probe tip.

There are several study limitations that are worth noting. Only three fiberoptic probes from one vendor were evaluated in this study, and only two of those probes with the most and least extreme VHA were evaluated in the tissue-mimicking phantoms. Testing with other probe types was not included and was considered beyond the scope of the work. In addition, this study only evaluated the performance of the fiberoptic probes at two FUS frequencies (1 and 3 MHz). Higher frequencies (>3 MHz) could alter the results, as probe response could be a function of both wavelength and how the material impacts the VHA.

## Conclusion

The thin-diameter (0.14 mm) glass fiberoptic probe with ethylene tetrafluoroethylene coating monitored FUS-induced heating in the acoustic field without evidence of a VHA. The ability to perform calibration and accuracy studies in an active acoustic field will broaden the range of testing allowed, improving the calibration range of MRTI sequences.

## Figures and Tables

**Table 1:** Fiberoptic probe types and characteristics with testing conditions evaluated including environment, acoustic incidence angle and acoustic intensity.

	Fiberopticprobe type		
	PRB-500	PRB-140	PRB-G40
**Accuracy (°C)**	± 0.1°C	±0.1°C	± 0.1°C
**Response time constant (ms)**	600	150	300
**Tip diameter (mm)**	1	0.14	0.75
**Tip material (sheath)**	Nylon	None up to 0.2 mm, then ETFE	Polyimide
**Tip material (fiber core)**	Polymer optical fiber	Glass optical fiber	Glass optical fiber
**Testing conditions**			
**Acoustic free-field testing**	X	X	X
Acoustic intensities (W/cm^2^)	250, 550, 1025,1610, 2300, 3125, 4100		
Frequency (MHz)	1, 3	1, 3	1, 3
**Tissue-mimicking phantom testing**	X	X	
Incidence angle	180°	45°, 90°, 180°	n/a
Acoustic intensities (W/cm^2^)	250, 550, 750	250, 550 (45°)	n/a
		250, 550, 750,1150 (90°) 250, 550, 750 (180°)	
Frequency (MHz)	1, 3 (2300 W/cm^2^)	1, 3	1, 3 (2300 W/cm^2^)

The response time constant is the time required for the temperature sensor to respond to approximately 63.2% of a sudden temperature change.

ETFE, ethylene tetrafluoroethylene.

## Data Availability

The data presented in this work will be made available upon reasonable request.
